# CFD modelling and simulation of anaerobic digestion reactors for energy generation from organic wastes: A comprehensive review

**DOI:** 10.1016/j.heliyon.2025.e41911

**Published:** 2025-01-10

**Authors:** Muhammad Usman Farid, Indiana A. Olbert, Andreas Bück, Abdul Ghafoor, Guangxue Wu

**Affiliations:** a Institute of Particle Technology (LFG), Department of Chemical and Biological Engineering, Friedrich-Alexander University Erlangen-Nuremberg Cauerstr, 4, D-91058, Erlangen, Germany; b Civil Engineering, School of Engineering, University of Galway, Galway, H91HX31, Ireland; c Department of Farm Machinery and Power, University of Agriculture, Faisalabad, 38000, Faisalabad, Pakistan

**Keywords:** *Anaerobic digestion*, *Reactor design*, *CFD*, *Hydrodynamics*, *Multiphase*

## Abstract

Anaerobic digestion (AD) has been recognized as one of the most viable options for the treatment of a wide range of waste materials. Complex structure of wastes is safely broken down to destroy pollutants and pathogens. Biogas is produced as a by-product of this process which is considered as a clean energy resource. However, provision of controlled environment for microbial activities is critical to ensure the required process efficiency. This can only be achieved with the efficient design of controlled vessels used for anaerobic digestion, termed as AD reactors. AD functions such as mixing, hydrodynamics, multiphase interaction, heat transfer, temperature distribution and bio kinetics are significantly affected by the reactor shape, design and configurations, hence making it essential to optimize the reactor design before installation at large scale. Mostly, such optimization is carried out with the help of lab scale experimentations and testing protocols which result in high costs for repeating several design experiments. Computational fluid dynamics (CFD) is an applied mathematical tool which helps to understand and predict the fluid dynamics, heat flow as well as species transport in different domains. This approach contributes to minimize the experimental costs while optimizing the reactor configurations in less time. The current review is presented to summarize and discuss the core characteristics of AD process followed by concerned CFD attributes. Research gaps and critical challenges are identified in different aspects such as reactor design, and configuration, mixing, multiphase flow, heat transfer, biokinetics as well as machine learning approaches.

## Highlights

• Overview of the core characteristics of AD process and their functions.
• Different designs and types of AD reactors are compared and discussed.
• Mixing and hydrodynamics in AD reactors and its impact on overall performance.
• Modelling and simulation of AD reactors through CFD approach is discussed briefly.
• Different models are compared for reactor design optimization through CFD.

## 1 Introduction

Booming population and urbanization have resulted in rapid increment in waste/wastewater generation, challenging sustainability of our society, economy, and environment. The current total global waste is being generated at a rate of 2.63 tonnes/capita/year, which is expected to reach 46 billion tonnes with a projected population of 9.7 billion by 2050 [[1], [2], [3]]. While global wastewater generation has been estimated to be approximately 365 × 10^9^ m^3^/year [4]. At the same time, fast growth of industrialization of various sectors has put significant burden on the available energy resources. Nowadays, with the control of carbon emissions, there is a need to introduce alternative ways to meet the energy demand. Introduction of renewable energy technologies has been recognized as a sustainable way not only to meet energy demand but also to achieve pollution control [5,6].

**Fig. 1:**
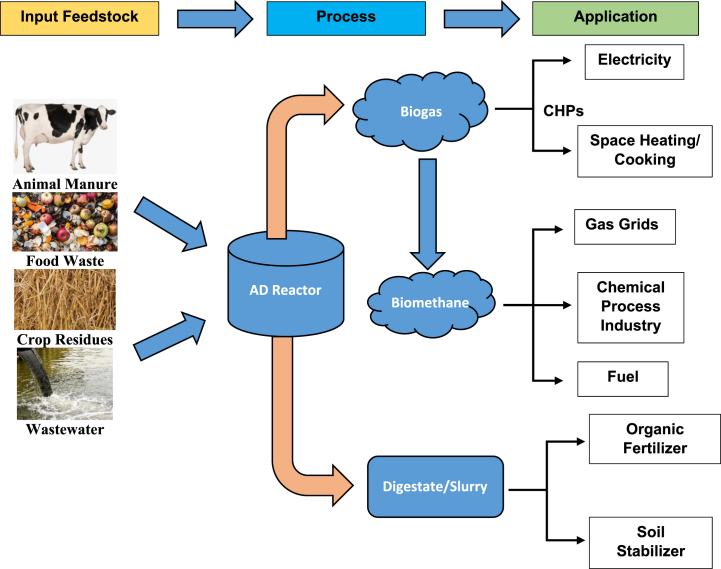
Application and benefits of AD process [8].

Anaerobic digestion (AD) has been recognized as one of the most viable bioenergy processes in which organic wastes are biologically degraded in the absence of oxygen. The feedstock organic wastes may be released from agriculture and dairy farming, food production and consumption as well as municipal activities. The complex structure of the organic matter is destructed by microorganisms and a gaseous mixture is released termed as biogas [3] which can be used as energy source. In some cases, the gaseous mixture can be refined to other value-added products for the process industry. While the slurry or digestate produced from anaerobic digestion consists of significant amount of nutrients, which can be used as an organic fertilizer with less environmental impacts as compared to the synthetic chemicals (Fig. 1) [[7], [8], [9]].

**Fig. 2:**
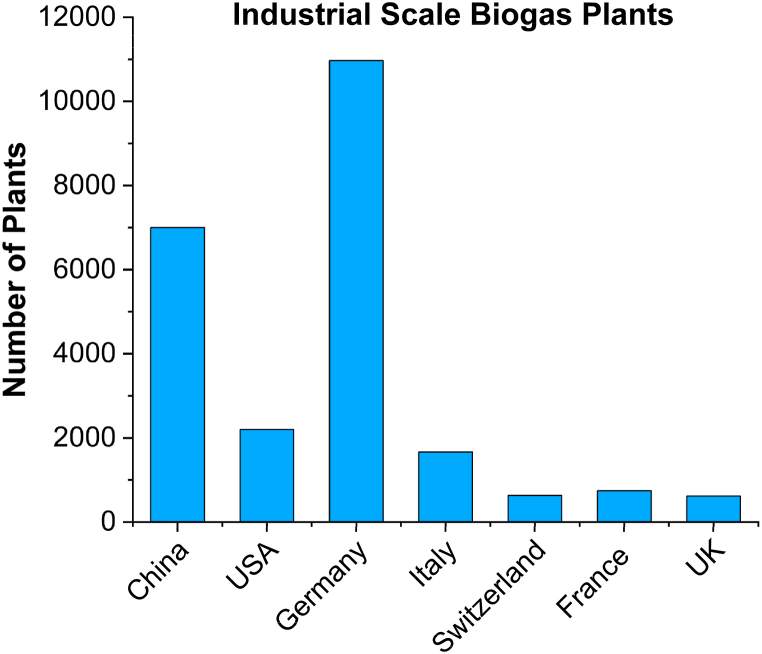
Industrial scale AD (biogas) plants [10].

During the last decade, 90 % increment in biogas technology has been reported showing an increasing interest towards this technology [10]. Though AD is a well-established technology and has gone through considerable development phases to understand and characterize the biological activities involved in AD process, the destruction rate of organic matter and the biogas yield are still a critical challenge. Anaerobic digestion has gained a significant popularity in industrial scale applications due to its natural biological process. Fig. 2 illustrates a comparison of industrial scale AD (biogas) plants among various regions. However, the design and configuration of different AD components is an overriding issue while considering large and industrial scale AD applications (Fig. 2) [7,11].

Being a natural process, suitable ambient and localized environmental conditions are required by biological agents to live, grow and perform biochemical transformation activities. Any change in such local conditions significantly affect the overall AD process. For example, temperature of substrate (i.e., organic feedstock), hydraulic retention time (HRT) and mixing of raw feedstock are one of the major characteristics which significantly affect the anaerobic digestion process and production of biogas. Moreover, different environmental conditions are needed to be kept constant in different phases of the fermentation process. Hence, provision of optimum conditions is the basic and uncompromising requirement for an AD process [12].

The design of reactor needs particular attention as the efficiency of AD phases greatly depends on its shape and geometrical configuration. Hence, selection of appropriate reactor design is a fundamental and crucial step in large scale AD systems [13,14]. Comprising of different processes, it is also essential to optimize the operating conditions with the novel AD reactor design to mitigate operational problems faced in large scale systems. A typical AD reactor is distinguished to have different processes occurring within the same domain, i.e., hydrodynamics, multiphase flow, heat and mass transfer and reaction kinetics. Numerical simulation with computational fluid dynamics (CFD) technique can be a useful tool to configure a suitable shape, geometry and dimensions of AD reactor. This provides an insight about the physical and biochemical processes occurring in the vessels. Similarly, this also contributes for further multiscale applications of the reactors at required scales.

**Fig. 3:**
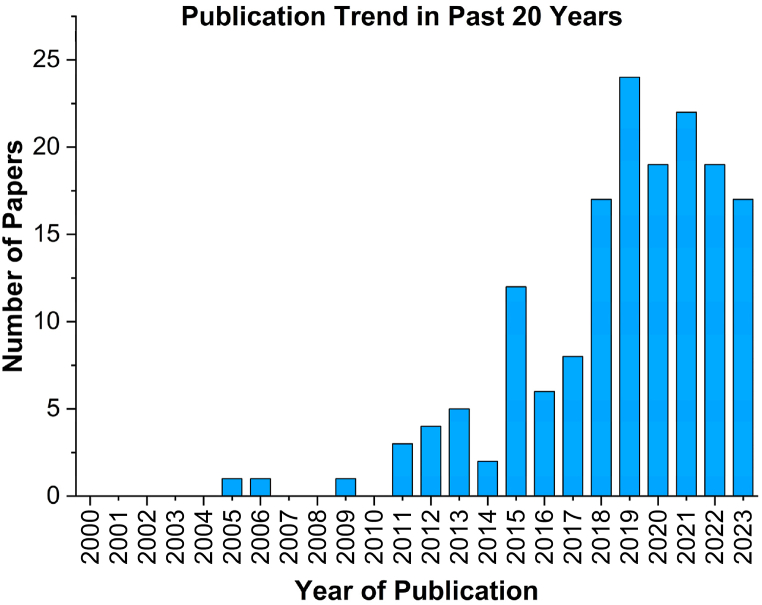
Bibliometric analysis of CFD simulation of AD process (web of Science®).

Fig. 3 shows the research trends in the field of CFD modelling and simulation of anaerobic digestion (AD). The bibliometric data from 01 to 01–2000 to 15-08-2023 was extracted from Web of Science® with keywords of ‘Anaerobic Digestion’ and ‘CFD’. A total of 161 publication are recorded with the most conducted in the recent decade (Fig. 3). This shows a significant attention of the research community towards this area. While only limited data is available with the clear guidelines to simulate anaerobic digestion (AD) reactor using CFD approach.

A few review studies have been presented on modelling and simulation of bioreactors using CFD in recent years. For example, Caillet et al. [15] reported advances in CFD modelling of AD process for energy production. Li et al. [16] studied and discussed the adoption of CFD modelling to investigate the mixing performance in the AD process. CFD applications were assessed by Sadino-Riquelme et al. [17]. Bastiani et al. [18] also presented a review on CFD modelling of granular sludge AD reactors. Although these studies have presented interesting aspects of CFD modelling of AD processes, they mostly focused on specific reactors types or specific processes in AD. Moreover, the linkage between physical processes/phenomena, design configuration and the respective CFD modelling strategy is still not fully addressed and understood. Therefore, this review aims to provide common fundamentals and core characteristics of all AD processes. This is followed by a CFD segment for design optimization of different AD reactors. For this purpose, this study is divided into two sections: (1) core characteristics of anaerobic digestion are identified; (2) CFD strategies to model these characteristics are presented. Reactor design and process configuration is discussed and guidelines for CFD modelling and simulation are presented.

## 2 Types of AD reactors and core characteristics of AD processes

### 2.1 Anaerobic digestion and bio-reaction

The destruction of the biodegradable fraction of organic waste is carried out in four different phases, i.e., hydrolysis, acidogenesis, acetogenesis and methanogenesis. During the first phase, the complex structure of organic matter is broken down to carbohydrates, protein and fats which are then further converted to sugars, amino acids and long chain fatty acids, respectively. During acidogenesis, the sugar and amino acids are destructed to formulate volatile fatty acids, i.e., acetate, propionate and butyrate etc. And some of them are then further fermented to acetate and hydrogen during the phase of acetogenesis. During the last phase, the methanogens split acetate into methane and carbon dioxide while hydrogen and carbon dioxide produced during the acetogenesis phase are also chemically reacted to form methane and carbon dioxide by hydrogenotrophic methanogens [8,19].

Organic matter exhibits a typical hydrocarbon structure comprising of carbon (a), hydrogen (b), oxygen (c), nitrogen (d) and sulphur (e) which undergo biochemical reactions to produce a mixture of methane, carbon dioxide and some portion of ammonia and hydrogen sulphide. Amount of these constituents can be determined thorough elemental analysis/ultimate analysis which provides a baseline theoretical estimation of the biogas production under AD process. For this purpose, a modified version of Buswell equation [20] was presented by Boyle [19,[21], [22], [23]]:

(1) $$C_{a}H_{b}O_{c}N_{d}S_{e}+\left(\frac{4a-b-2c+3d+2e}{4}\right)H_{2}O=\left(\frac{4a+b-2c-3d-2e}{8}\right){CH}_{4}+\left(\frac{4a-b+2c+3d+2e}{8}\right){CO}_{2}+{dNH}_{3}+{eH}_{2}S$$

### 2.2 AD reactors

**Fig. 4:**
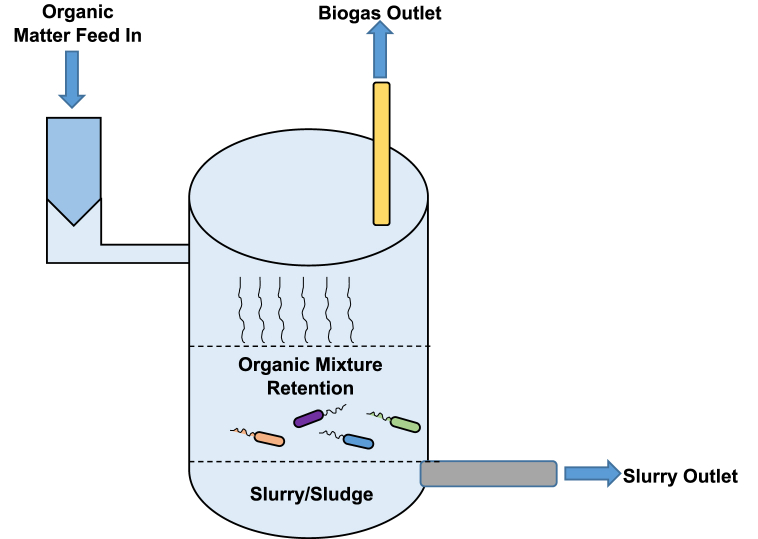
Components of a typical AD reactor.

Anaerobic digestion is carried out in controlled atmosphere, i.e., AD reactors (mostly in a cylindrical form), which can provide favourable environmental conditions for the microorganisms to perform biodegradation processes. A typical AD reactor has three sections, i.e., the biological reaction chamber, the three phase separation section and the gas/slurry/sludge outlet (Fig. 4). The biological reaction chamber is used to retain the organic matter for a specific period of time termed as hydraulic retention time (HRT) during which microorganisms destruct the organic matter to produce the methane and carbon dioxide mixture (biogas). After separation, as shown in Fig. 4, biogas is collected from the gas outlet and treated liquid flows out in the form of effluent (mostly in case of wastewater), while maintaining sufficient solid contents in the reaction chamber, a part of semi-solid matter is discharged through the slurry outlet [24].

Reactor shape and design configuration affect microbial activities through influencing the hydrodynamic behaviours of substrates inside the reactors. Temperature and other local environmental conditions also be affected by the shape, size and configuration of bioreactor [25]. The volume of reactor domain has a direct relation with the HRT and can be estimated from Refs. [26,27]:

(2) $$RV=HRT\times \frac{DFI}{\rho_{s}}$$

where, RV is reactor volume (m^3^), DFI is daily feedstock intake (kg/day) and $\rho_{s}$ is density of substrate in the digester (kg/m^3^). A margin of 25 % is provided in the reactor volume for the collection of gas in the upper region, hence the total volume of reactor is RVT = RV + 0.25 × RV) [26,27].

**Fig. 5:**
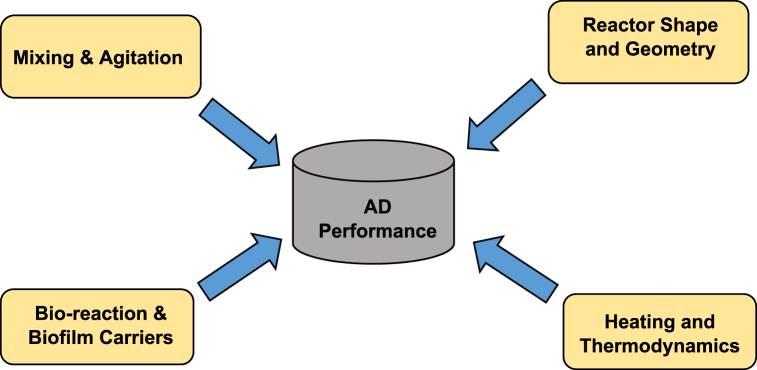
Core characteristics of AD process.

Although considerable efforts have been carried out in order to improve the anaerobic digestion process, the overall efficiency of the system is still a question for many applications. In order to improve the AD process efficiency, it is essential to understand the key performance parameters (Fig. 5) which are directly linked with the biodegradation process of organic wastes into biogas.

The design, shape and configuration of AD reactor has gone through a revolutionary phase to improve the process efficiency. Mainly two groups of AD reactors have been developed, i.e., low-rate reactors and high-rate reactors. Former category includes fixed dome, floating drum and bag type/plastic drum reactors while later group of AD reactor includes up flow anaerobic sludge blanket (UASB), expanded granular sludge bed (EGSB) and internal circulation (IC) reactors. Table 1 shows a comparative summary of different versions of AD reactors in terms of advantages and limitations. Fixed dome reactor is one of the oldest designs of AD reactors in which the main digestion unit is typically installed below the ground. Feedstock inlet as well as slurry outlet is attached in such a way that both are dipped within the digestion chamber to avoid leakage of biogas. Having low heat losses, such reactors are mostly suitable for the regions with cold climate. By estimation, 25 kg of manure can produce of 1 m^3^ biogas in the fixed dome reactor. Because of irregular gas pressures generated in the digestion chamber, regular monitoring is required to avoid damages of the reactor [26,27].

**Table 1:** Comparison of different versions of AD reactor [18,[25], [26], [27], [28], [29], [30]].

Reactor Type	Technical Expertise	Construction Cost	Operating Cost	Advantages	Limitations
Fixed Dome Reactor	Low	Low	High	Simple construction High life span Low heat loss Applicable to cold regions	Risk of damage due to high pressure Regular monitoring required
Floating Drum Reactor	High	High	Low	Bearable to high pressure Easy monitoring of gas production	Complex structure Leakage of gas Long HRT Heating required
Bag Type Reactor	Low	Low	Low	Shallow construction Easy cleaning Easy monitoring of gas production	Low gas pressure Pumps required Short life span External heating is difficult
Plastic Reactor	Low	Low	Medium	Portable construction On-site waste treatment	Low efficiency Low gas pressure Not applicable to large scale treatment
UASB Reactor	High	High	Low	High Process efficiency No external mixing needed	Complex and technical construction Inoculation required
EGSB Reactor	High	High	Medium	More particle interaction Applicable to diluted waste streams	Complex and technical construction Inoculation required Drainage of particles with outgoing stream
IC Reactor	High	High	Medium	High treatment and gas removal efficiency Recirculation of sludge	Expertise required for construction Mechanical treatment required to crush large organic matter to fine particles

Floating drum reactor is another design of AD reactors in which an inverted cylindrical drum is provided over the digestion chamber to collect the produced biogas through AD process (Table 1). This gas collection drum is designed based on the floating mechanism which can bear greater pressure and volume of gas compared to the fixed dome reactors. Better inherit mixing is another advantage of such reactors. However, leakage of biogas from the floating drum is a major limitation for such design of AD reactor. Moreover, development of floating drum reactor is more complex and requires higher initial investments than the fixed dome reactor [28].

Bag type (also termed as balloon type) is another version of AD reactors in which a bag or balloon acts as the digestion unit. The gas collection outlet is attached to the upper part of the bag while inlet and outlet for substrate and slurry are connected on both sides of the bag. This makes it easier to handle and operate for distinct locations. Due to shallow construction, such reactors can be installed at areas having water-logging due to an increased ground water table. Shorter life span (2–5 years) and low gas pressure are some of the major limitations of such reactors (Table 1) [29]. Plastic AD reactors can be easily constructed from polymer materials such as PE, PVC, HDPE, PP, and other reinforced plastics. Due to lower efficiencies and gas production, such reactors are better suitable for the small-scale household application rather than large-scale AD systems [29].

From the category of high-rate AD reactors, up flow anaerobic sludge blanket (UASB) reactor is one of the modern AD reactors (however, the concept was introduced during 1970–80s) in which the digester is divided into three different zones. The bottom zone consists of packed layer of high density bio-solids (sludge) termed as sludge bed zone. The zone above the sludge bed zone is a suspended layer of water, solids and gas named as the sludge blanket zone while the upper zone of the reactor is termed as 3-phase separator zone and used to separate the gas bubbles from the water and solids regime in the reactor. Mixing of the substrate in UASB is achieved through biogas perturbation and the water up-flow. With a better mixing and interaction of organic matter with the bacterial species, UASB possesses a higher degradation efficiency than the conventional AD systems [18]. Expanded granular sludge bed (EGSB) reactor is developed as an improved version of UASB reactors. Organic solids (sludge) in EGSB reactor are better uniformly dispersed in the digestion chamber rather than packed in the bottom by introducing a high speed jet of recycled gas or liquid. This also introduces the formation of granules within the EGSB reactor and hence enabling long solids retention time [30]. Internal circulation (IC) reactors are another modified design of UASB reactors in which 3-phase separator zones are formulated in different heights in the reactor to act as a static mixing mechanism. A pipe carries the gas and bubbles to the upper unit of the reactor from which gas is removed and slurry mixture is returned to the digestion chamber making an internal loop of circulation. This enables more efficient digestion of the organic substrates, hence regarded as the 3rd generation reactors. However, dispersion of solid particles and its interaction with the other phases makes it essential to study and optimize the hydrodynamic behaviours in the novel reactors (Table 1) [18,30].

### 2.3 Mixing of organic substrates

Organic substrates such as wastewater fed into the AD reactor consist of floating solids, i.e., fibre, straws etc. which float on the liquid surface as well as heavy solids which usually settle down in the vessel bottom during the retention time. Floatation of solids result in a dense layer on the liquid/substrate surface and cause hindrance in release of biogas bubbles. On the other hand, a thick sludge layer is formed in the vessel bottom causing mechanical faults of the system. In both cases, active volume in the reactor is decreased and thereby interaction of the organic matter with microorganism and nutrients is also affected eventually reducing the process efficiency. Hence, it is necessary to agitate the substrates to enable suspension volume in the reactor which can be carried out using ‘mixing’. Hence, mixing is one of the fundamental physical factors which directly affects the efficiency of microbial processes such as in anaerobic digestion. Uniform temperature and homogenous pH of the entire volume, efficient heat and mass transfer, reduction of scum and crust formation, prevention of floc formation, proper interaction of microorganisms with the organic matter and nutrients as well as easy release of biogas bubbles are some of the major advantages of mixing in AD process [31]. Digestion failure may occur due to inadequate mixing while extensive mixing disturbs the microbial species in the AD reactor. This necessitates the need of optimum mixing in the AD reactor. Mixing of substrates in AD reactors can be achieved through different approaches such as mechanical mixing, pneumatic mixing and hydraulic recirculation, as discussed below.

#### 2.3.1 Mechanical mixing

Mechanical mixing is a common practice used in the completely mixed anaerobic reactors where a mechanically powered mixer/stirrer is used to agitate the mixed liquor with the possible TS 3–10 %. The organic mixture is feed in and out in a continuous manner and the quantity of substrate mixture is maintained in the digestion chamber. With continuous mixing, the composition of the substrate can be easily kept uniform within the AD reactor. This leads to steady production and supply of biogas from the reactor [32,33].

**Fig. 6:**
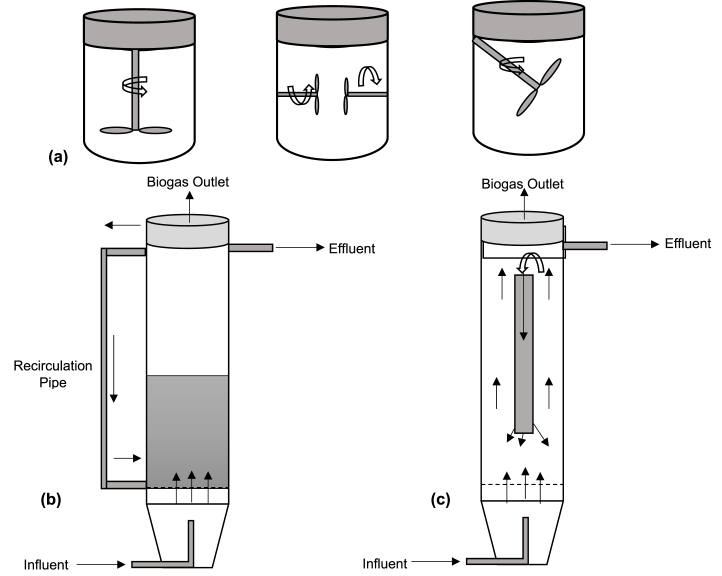
Different mixing strategies **(a)** mechanical mixing **(b)** gas recirculation **(c)** liquid internal recirculation [18,37,40,42,43].

A few mechanical agitation approaches are applied to AD process depending on the design of shaft with number of blades and propellers as well as installation position (Fig. 6 (a)). Moreover, operating strategies such as rotational speeds, operating frequency (continues or intermittent) and mixing time also effect the mixing efficiency [34]. It has been reported that around 29 % of the operating cost can be saved by using intermittent mixing, however, operation optimization is needed for a certain kind of substrate to ensure suitability of AD process [35].

Fluid rheology is a key factor when selecting an appropriate design of mechanical mixing system for anaerobic digestion. The design configuration of agitators and mixers is a critical stage and has been investigated intensively for AD reactors. For example, Wu [36] found that ribbon bladed agitators work on the principle of axial mixing mechanism in which pumping flow depends upon the rotation direction of the mixer, while agitators with anchor blades give a radial flow field which is independent of rotational direction. Wiedemann et al. [37] investigated different design of mixers in biogas reactor, i.e. 3-bladed small propeller (SP), 3-bladed big propeller (BP) and 4-bladed paddle mixer (PDL). With these mixer designs, three combination of side mounted submersed variable positioned configurations were studied, i.e., SP-BP, BP-BP and BP-PDL. It was concluded that installation of multiple mixers at different heights result in more uniform mixing outcomes. However, mixers installed with top entrance configuration provide better mixing but lead to more operating cost (Fig. 6 (a)). With optimized mixing performance, around 30 % of the operating cost can be reduced. Similarly, Rasouli et al. [38] studied 32-bladed radial mixer in a plug flow reactor. In this case the substrate is mixed in radial direction throughout digestion chamber while biogas generated is collected from the outlets provided at the top of horizontal channel [32].

With several advantages of mechanical mixing, it is also important to consider the power requirements for agitator. It has been estimated that mixing of substrates accounts for approximately 50 % of the total input energy [37]. Hence, it is important to improve the mixing efficiency in line with the power input. The fluid rheology, particularly TS has a prime importance while estimating the power input.

The Reynolds number ($R_{e}$) for the non-Newtonian fluids mixed with the help of a mechanical agitator is given by Ref. [36]:

(3) $$R_{e}=\frac{\rho_{s}Nd^{2}}{\eta_{s}}$$

where, $\rho_{s}$ is density of substrate inside the AD reactor, $\eta_{s}$ is the viscosity of substrate, N and d are the speed and diameter of the agitator/mixer, respectively.

The power ($P_{m}$) required by agitator (W) can be calculated from Ref. [39]:

(4) $$P_{m}=2\pi N\tau_{m}$$

where, $\tau_{m}$ is the torque (Nm) applied to the agitator/mixer.

Power number $N_{p}$ for the agitator/mixer is given by Refs. [36,40,41]:

(5) $$N_{p}=\frac{P_{m}}{\rho_{s}N^{3}d^{5}}$$

The torque and mixing speeds can be measured experimentally to calculate power requirement for an agitator/mixer. It should be noted that the above expressions can be used to estimate the power requirements on the given speed of an agitator while flow regimes inside the reactor cannot be described to improve mixing efficiency. However, the information obtained from these empirical correlations can be used to validate the results obtained from CFD simulations.

Although mechanical mixing is a common practice but this technique faces certain challenges while handling high solid contents. More power is required to mix the high dense fluids and hence increases the operational cost. Technical expertise is required for regular cleaning and maintenance of agitator components. In some cases, overhauling of reactor is required for this purpose leading to high maintenance cost.

#### 2.3.2 Pneumatic mixing

Circulation of gas is another mixing approach used in the AD reactor which is applied to most of the moderate mixing applications, termed as pneumatic mixing. In this approach, biogas recirculation in the reactor causes internal turbulence to homogenize the substrates/mixed liquor (Fig. 6 (b)). This involves simpler configuration of the AD reactor as compared to the mechanical mixing which consist of complex structures of moving parts [44]. Besides uniform mixing of substrates, biogas recirculation also enhances the bio solids/sludge mobilization as well as dissolution of carbon dioxide and hydrogen in liquid phase to enrich methane content in the gas phase. Moreover, it helps to release the trapped biogas bubbles in the sludge [45,46]. In some cases, hydrogen gas is also used as a recirculation medium to upgrade biogas. For example, Khan et al. [47] found that 99 % methane contents could be obtained by recirculating the biogas and hydrogen mixture at a rate of 32 L per min for 12 h duration. Despite of several advantages of biogas and hydrogen recirculation, operational safety is a critical challenge. Moreover, insufficient biogas levels may also result in non-uniform mixing. Furthermore, employing air instead of biogas for mixing of AD reactor was reported by Yang and Deng [48]. Because certain anaerobes can tolerate oxygen/air during the injection of limited amount of air to achieve the micro-aerobic environment could enhance mass transfer rate, resulting in approximately 6 % and 12 % more methane production compared to biogas recirculation and a mechanical mixing, respectively. The physics of gas mixing is a complex process which involve three phase interaction and hence very challenging to characterize its hydrodynamic behaviour.

#### 2.3.3 Hydraulic recirculation

Hydraulic recirculation of liquid/slurry in the AD reactor is applied to the feedstock with less TS concentrations, i.e., wastewater which consist of suspended particles (Fig. 6 (c)). Internal hydraulic recirculation can be carried out either by applying high mixed liquor flow rate causing internal fluidization (i.e. EGSB reactor) or recycling the separated liquid after the three phase separator zone and injecting back to the internal reactor domain again to mix the new incoming substrates (i.e. IC reactors) [18]. Compared with other mixing technique, hydraulic recirculation provides several advantages such as minimum risk of gas leakage, low explosion risks, flexibility in operation as well as easy maintenance [42,48]. Similarly, this approach also helps to dilute the influent and hence, makes it feasible to treat toxic compounds. However, a high velocity of fluid may wash-out the sludge particles from the reactor (Fig. 6 (c)). Due to this reason, it is extremely essential to optimize the fluid recirculation velocities which can efficiently fluidize the biomass particles while maintaining the required sludge concentration for stable anaerobic process [49].

### 2.4 Heating and thermodynamics in AD

AD process is a biological fermentation process governed by microbial species (bacteria) which exist in a specific temperature range. The temperature influences the growth and metabolism rate of microorganisms which eventually effect the overall AD performance (Fig. 5). Table 2 shows the temperature range required by the bacterial groups to perform the AD process. Psychrophilic category includes the lowest temperature range i.e., 10–20 °C which implies that this process can be operated at low temperature regions. However, the digestion process may take extremely long time making it unsuitable for large scale systems. Mesophilic range (20–40 °C) is most commonly applied to a wide range of organic matters, however external heating is required for the region with cold climate. Similarly, AD process can be excited by using thermophilic and hyper-thermophilic group with temperature ranges 40–60 °C and 70–80 °C, respectively (Table 2). Pathogens present in the organic substrate can be easily destroyed at such high temperature. Moreover, organic load with a high initial temperature (i.e., industrial wastewater etc.) can be treated easily without cooling it to a further lower temperature for other bacterial groups. However, a slight fluctuation from the described range may result in unstable AD process and slowing the bacterial activities. With such sensitive temperature requirement, heating and temperature distribution should also be studied while designing and optimization of AD reactors [42,[50], [51], [52], [53], [54], [55], [56]].

**Table 2:** Temperature requirement for different AD approaches [42,[50], [51], [52], [53], [54], [55], [56]].

Operation Category	Range	Advantages	Limitations
Psychrophilic	10–20 °C (Usually 20 °C)	• Low Cost • Less Energy Input • Applicable to Cold Regions	• Low Efficiency • Unstable and Very Long HRT • Small Scale Usage
Mesophilic	25–40 °C (Usually 35 °C)	• Process Stability • Resistance to Toxicants • Wide Application Range	• Long HRT • Require Large Volume • Risk of Foaming
Thermophilic	40–60 °C (Usually 45 °C)	• High Metabolic Rates • Ability to Disinfect Pathogens • Less Start-up Time • Short HRT	• VFAs Accumulation • High External Energy Input • Sensitive to Temperature Variation
Hyper-thermophilic	70–80 °C (Usually 70 °C)	• High Conversion Rates • Treatability to Organic Wastes with High Temperature (Industrial Effluents) • Effective Soluble to Non-Biodegradable Fractions	• High Energy Input • Sensitive to Temperature Variation

### 2.5 Biofilms carriers as biocatalyst

Application of biofilm carriers to the AD reactor helps to maintain the essential microbial populations with low growth rates. This also helps to tackle the wash out of core biomass material from the AD reactor. Granular or fibrous carriers can be applied for this purpose depending on their material characteristics [57]. However, interaction of such discrete particles (explained later in section 3.2) with other phases, i.e., liquid and gas in AD reactor has a great influence on reactor hydrodynamics and overall process efficiencies, which deserve deep investigations.

## 3 CFD modelling of AD process

AD reactors act as biological treatment units where geometrical configuration, inflow and outflow of substrates, hydraulic retention, multiphase patterns and interaction of species significantly affect the overall process efficiency. For a general perspective, three phases are formed in a typical AD reactor, i.e., solid phase (slurry/sludge), liquid phase (influent containing colloidal) and gas phase (biogas). For an efficient and cost effective process, it is extremely important to optimize the reactor configuration in line with the process parameters. For this purpose, lab scale experiments are conducted, conventionally, to mimic the AD process at large. However, it has been found that such small scale experimental tests are based on empirical relations whereas hydrodynamic behaviours (i.e., density of fluid, mixing and interaction of solid particles) deviate significantly while considering the large scale systems. Moreover, experimental techniques for AD process are highly time consuming, unattainable for micro level fluid dynamics and limited by sophisticated lab equipment requirements [18,58].

**Fig. 7:**
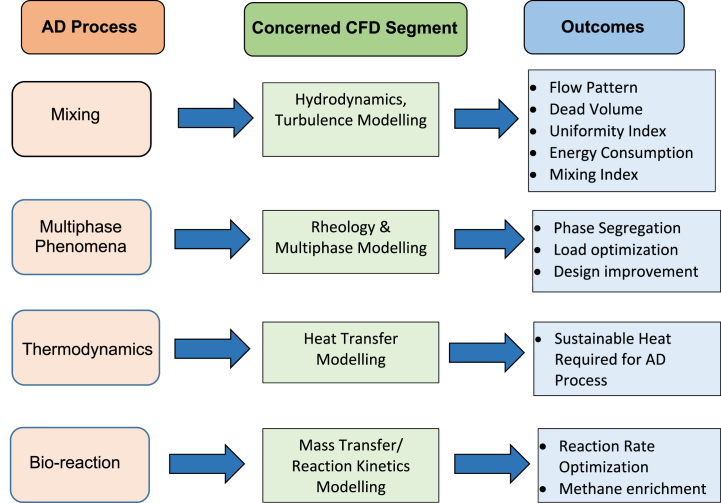
CFD modelling attributes for AD process.

Additionally, it is also important to study the theoretical principles of anaerobic digestion process to judge the accuracy of experimental findings. Having a closed loop system with input and output species, AD process can be modelled to predict possible products from the biochemical degradation of organic matter. CFD modelling of AD reactor can be divided into different segments, i.e., hydrodynamic modelling, multiphase modelling, heat transfer modelling and bio-kinetics modelling approaches (Fig. 7).

**Fig. 8:**
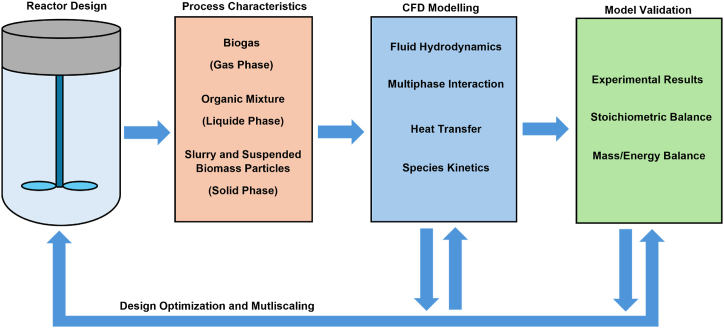
Design Optimization of AD Reactor using CFD Approach.

ADM1 model was presented by Batstone et al. [59] which integrates the biochemical reactions and the operating conditions such as organic substrate composition, pH, temperature and the resulting products of the biochemical reaction. The model has been found to be accurately predict the AD process outcomes which correspond to the experimental tests. However, this model has certain limitations, i.e., the substrate is assumed to be uniformly mixed in the reactor domain which neglects the dead zones formed in the reactor because of poor mixing. Similarly, the model can be solved for different operating conditions, however, the variations in the reactor domain is compromised which dominantly affect the reactor configuration [18]. Computational fluid dynamics (CFD) is one of the applied mathematical techniques which helps to understand the hydrodynamic behaviour of fluids in a given system (Fig. 7). CFD comprises mathematical codes and algorithms used for the solving different governing equations for fluid flow, momentum, energy and mass transfer as well as species transport and conversions (Fig. 8). The codes used to solve CFD problems include ANSYS (Fluent), OpenFOAM, STAR-CCM + etc., adoption of either package depends on the available resources and computation power [60,61].

Fig. 8 represents the linkage between the AD core characteristics and the concerned CFD segments for a specific design of AD reactor. Design of the reactor is a primary factor which greatly influences the internal flow pattern and hydrodynamics (Section 2.3), therefore, modelling of fluid flow is the foremost step in CFD. The momentum caused by multiphases (i.e., wastewater, sludge particles, biogas bubbles) significantly contribute in mixing, internal recirculation as well as degradation efficiency, hence, inclusion of multiphase (sub) model is inevitable. Relying greatly on the temperature (Section 2.4), the modelling of heat transfer helps to optimize the reactor and its operating conditions. Additionally, modelling of species kinetics also helps to predict the degradation rate and formation of multiphase concentration in the reactor. Hence, a comprehensive CFD model for design optimization and simulation of AD reactor includes following segments:

- Modelling of fluid flow and hydrodynamics
- Modelling of multiphase behaviour
- Modelling of heat transfer
- Modelling (and coupling) of species kinetics

The application and adoption of these sub-models in CFD are explained as below:

### 3.1 Modelling of fluid flow and hydrodynamics

Although organic matter is retained in the digestion chamber for a specific time period to be biologically degraded by microorganisms, the main digestion chamber experiences variable hydrodynamic trends due to inflow and outflow of the organic matter. Moreover, the gas bubble formation, release and movement also cause localized turbulence in different locations inside the reactor. This phenomenon is more critical for the case of modern AD reactors, i.e., high rate up flow reactors (section 2.4). Moreover, mixing is one of the major factors influencing the digestion efficiency. This results in proper interaction of the particles with microorganisms, maintaining the pH in the whole volume and reduces the inhibition of dead volume in the reactor. An agitation in the digestion is carried out by using mechanical mixers and agitators which cause formation of active volume of the fluid within the domain with a Reynolds number exceeding 20,000 hence can be regarded as turbulent flow which needs to be modelled. Besides improving the AD process, mixing and agitation of AD reactors increase the operational costs in terms of input energy. Hence, an optimum mixing speed is required for profitability of AD process. This can be achieved by modelling the mixing characteristics of desired organic matter in the AD reactor domain using CFD (Fig. 8). Various turbulence and flow fluid flow models are available which can be used to solve turbulent regimes in the desired domain (Table 3) [35,40]. Choosing an appropriate fluid flow (turbulence) model is necessary to describe the flow fields accurately. Among different modelling approaches (DNS, LES etc.), eddy viscosity model is economically feasible approach which is based on RANS (Reynolds Averaged Navier-Stokes) equation. The fundamental equation for the averaged continuity/mass conservation and momentum is given by Refs. [[62], [63], [64], [65], [66]]:

(6) $$\frac{\partial }{\partial x_{i}}\left(\rho \bar{u_{i}}\right)=0$$

(7) $$\frac{\partial }{\partial t}\left(\rho \bar{u_{i}}\right)+\frac{\partial }{\partial x_{j}}\left(\rho \bar{u_{i}}\bar{u_{j}}+\rho \bar{u_{i}^{\prime }u_{j}^{\prime }}\right)=-\frac{\partial \bar{P}}{\partial x_{i}}+\frac{\partial {\bar{\tau }}_{ij}}{\partial x_{i}}$$

Where, ρ and P represent the Reynolds averaged velocity, density and the pressure whereas ${\bar{\tau }}_{ij}$ is the mean stress tensor.

**Table 3:** Application of RANS based fluid flow models [64].

Fluid Flow Models	Description and Application
Laminar	• Assumption of smooth and steady flow • Suitable for laminar flows
Spalart-Allmaras	• Comprises of single transport equation model • Suitable for aerodynamics applications • Challenging with complex flows under high pressure, i.e., formation of negative vortices and wake regions
k-ɛ Standard	• Baseline (default) 2 equation model in which coefficients are empirically derived • Assumption of the fluid as fully turbulent • Robust with wide application of fully turbulence flows, parametric studies and initial screenings
k-ɛ RNG	• A modified version of k-ɛ Standard model in which coefficients are analytically derived. • Suitable for shear flows with moderate swirls
k-ɛ Realizable	• A variant of Standard model with more accuracy as compared to RNG model • Challenging in case of multiple frame reference
k-ω Standard	• Baseline (default) k- ω model • Suitable for wall bounded, compressible and low Re flows
k-ω SST	• A variety of k- ω model Standard model with both near the wall and away from wall flows
RSM	• Capable of direct solution of transport equations • Computationally expensive • Suitable for complex 3-D flows with high swirling effects

Table 3 summarizes the different RANS based turbulence models and their applications. While all the model can be applied for the simulation of fluid flow in controlled structures, the accuracy and complexity of solution is always an important concern which can be handled with good agreement with the experimental results. Several researchers have used these turbulence models to simulate fluid flow in AD reactors (Table 4). For example, Wu [67] investigated six turbulence models available in ANSYS (Fluent) for the non-Newtonian fluids in an AD reactor and concluded that the k-ω (Standard) and k-ɛ (Realizable) models are the most suitable models for the simulation of mixing process in AD reactor. However, this study was based on single phase hence effect of gas bubbles was neglected which may affect the mixing trends. Moreover, he also recommended that configuration of two impellers with side walls of the reactor gives better results for the mixing of AD chamber. Later, Zhang et al. [40] studied the power consumption of mixers and fluid turbulence in the co digestion process with five different models and found that k-ɛ (Standard) perform with minimum error as compared to the other fluid flow models. Similarly, Sindall et al. [68] studied flow pattern in a cylindrical digester to find the appropriate sampling locations. They investigated four models, i.e., k-ɛ (Realizable), k-ω (Standard), k-ω (SST) and RSM to simulate the flow field in the reactor domain at different seeds of the agitator. It was concluded that k-ɛ (Realizable) fits better with the PIV results. Based on CFD simulation result, it was also recommended that sampling of the organic substrate should be collected near the impeller to assess the effect of mixing speeds on microbial species. Dabiri et al. [69] simulated a full scale AD reactor based on the argument that k-ɛ (Realizable) model has minimum computation power requirement as compared to the other models. The k-ω (Standard) was used by Wiedmann et al. [37] to investigate the mixing trends of AD reactor with different type of mixers. Latha et al. [70] used k-ɛ (Realizable) to study the mixing trends in lab scale AD reactor caused by hydrogen injection. Bridgeman [39] compared five turbulence models (with multiple reference frames) for mixing of sewage sludge in AD reactor and found that although RSM (Reynolds Stress models) gives less error but at the same time more computational power is required to converge the solution (Table 4). Moreover, he also recommended to couple the biogas yield model with CFD turbulence models for more realistic outcomes.

**Table 4:** Summary of CFD modelling approaches for AD reactors.

Bioreactor				CAD Domain and Meshing			CFD Approach for AD							MV	Ref.
Reactor Type	Size and Dim. of Reactor	Type of Mixer	Feed-stock	Domain (3D/2D)	Mesh Size	Grid Std.	Phase	Multi-phase Model	Turbulence Model	HT Model	Solver State	CT	Software Package		
Fixed Dome	H 0.92 m, R 1.219m, V 6 m^3^	Self-Mixing	Cattle Manure	3D	Tetrahedral 568046	Yes	L-G	E-E VOF Non-Newtonian Power Law	Laminar	–	Transient with time step 0.01 s	1–3 days	ANSYS (Fluent) V. 16.0	No	[71]
Pre-fabricated Type	H 1.27 m, R 1.09m, V 1 m^3^				Tetrahedral 643916										
Plug Flow Reactor	L 2.62 m, W 0.5 m, H 0.762 m V 1 m^3^				Tetrahedral 1013300										
Lab Scale Cylindrical Digester	H 200 mm D 200 mm	Four Flat Bladed Impeller	Sludge	3 D	1700000	Yes	L	–	Four Models a	–	Steady State	–	ANSYS (Fluent)	Yes	[60]
Lab Scale Digestion Tank	L 6.7 m D 12 m	Pitched Blade Turbine Impeller	Manure Slurry	3D	96460	Yes	L	–	6 Models^a^		Steady State	1 h	ANSYS (Fluent) V. 12.0	Yes	[67]
Stirred Anaerobic Digestion (AD) Tank (Full Scale)	H 3 m D 15 m	Submersible Rotating Mixer	Cattle Manure TS 12.1 %	3D	Polyhedral 222058	Yes	L (with TS)	Non-Newtonian Power Law	k-ɛ RNG	–	Both Steady State Transient	NR	ANSYS (Fluent) V. 19.2	Yes	[69]
Cylindrical Digester (Downscaled with a factor of 12)	H 0.7 m D 1.5 m V 1.2 m^3^	3 Mixers with side entry SP, BP and PDL	Biomass Substrate	3D	250000	Yes	L with TS	Non-Newtonian Power Law	k-ω Standard	–	Steady State	NR	StarCCM+	Yes	[37]
Lab Scale Cylindrical Vessel	H 0.2 m D 0.2 m	Four bladed flat impeller	Sludge	3D	Hexahedral 1409000	Yes	L	Non-Newtonian Power Law	k-ɛ Realizable, k-ω Standard, k-ω SST, RSM	–	Steady State	–	ANSYS (Fluent) V. 13.0	Yes	[68]
Lab Scale Cylindrical Reactor Tank	H 0.3 m D 0.115 m	Hydrogen Gas Jet with 10 mm injection	Leather Fleshing + MSW	3D	Hex/wedge 53170	No	L/G	E-L DPM + Power Law	k-ɛ RNG	–	Transient 0.05 s	–	ANSYS (Fluent) V. 6.3	No	[70]
Stirred Tank Reactor	H 0.32 m D 0.2 m	Mechanical Vertical Shaft with three layered pitch blade	Cattle Manure and Corn Stover (Co-digestion)	3D	742593	No	L/L	Two Phase Mixture Model	4 Models^a^	–	Steady State	–	ANSYS (Fluent) V. 14	Yes	[40]
Cylindrical Vessel	H 0.305 m D 0.160 m V 0.006 m^3^	2 six blade paddles	Sewage Sludge	NC	316704	Yes	L with TS	Power Law	5 Models	–	Steady State	4–8 h	ANSYS (Fluent) V. 6.3.26	Yes	[39]
Egg-Shaped AD Reactor	H 0.6 m D 0.13 m V 7.6 L	Self-Mixing	Wastewater Sludge	3 D	Tetrahedral 14412	No	L	Step Function Single Phase	Laminar	–	Transient		COMSOL V 4.4	Yes	[72]
Chinese Dome Digester	H 30.56 cm D 25 cm V 15 L	Self-Mixing	Liquid Slurry	3 D	Tetrahedral 160808	Yes	L	–	Laminar	–	Steady State	–	ANSYS (Fluent) V 19.2	Yes	[73]
Novel Prototype Plug Flow Reactor	D/L 1:5 V 1.35 m^3^	Flat Bladed Turbine Impeller	Cow Dung Diluted 88 % W 22 % D	3 D	Tetrahedral 1 × 10^6^	Yes	L/G	E-E Two Phase	k-ɛ RNG	–	Transient	–	ANSYS (Fluent)	Yes	[38]
Medium sized AD	H 6.7 m D 12 m V 791.28 m^3^	Gas Mixing with Gas Draft Tube	Liquid Manure (Slurry)	3 D	Hexahedral 52836	Yes	L/G	E-E Two Phase	12 Models	–	Unsteady	–	ANSYS (Fluent) V 12.0	Yes	[44]
UASB Reactor	H 2.12 m D 0.30 m V 140 L	Up Flow of Gas	Water and Exp. Gas	3 D	528000	Yes	L/G	E-E Two Phase	Laminar	–	Transient	–	ANSYS (Fluent) V. 16.2	Yes	[74]
Plexiglass Double Layer Reactor	H 590 mm D NM V 20 L	Up Flow of Gas Bubbles	Food Waste	3 D	Tetrahedral 265836	Yes	L/G	E-E Two Phase Power Law	k-ɛ RNG	–	Steady State	–	ANSYS (Fluent) V. 17.0	Yes	[75]
Commercial Anaerobic Digester	H 6 + 3 m D 25 m	Self-Mixing (Pumped Recirculation)	Sewage Water	3 D	Tetrahedral NM	No	L	NC	k-ɛ Standard	–	NC	–	ANSYS (Fluent) V. 16.0	No	[76]
Digester with Draft Tube	D 13.5 m V 1250 m^3^	Triple Helix Blade Impeller	Sewage Sludge	3 D/2 D	23 million cells	Yes	L with TS	Hershel-Bulkley Law	k-ω SST	–	Steady State	1 week	ANSYS (Fluent) V. 13,14,14.5	No	[77]
EGSB Reactor	H 120 cm D 63 cm V 3.35 L	Self-Mixing	Wastewater	2 D	5544	Yes	L/S/G	E-E Three Phase	k-ɛ Standard	–	Steady State	–	ANSYS (Fluent) V. 6.3	Yes	[58]
EGSB Reactor	H 1000 mm D 90 mm V m^3^	Self-Mixing	Wastewater	2 D	41536	Yes	L/S/G	E-E Three Phase	k-ɛ Standard	–	Unsteady	–	NM	Yes	[49]
Ontinyet-Agullent WWTPs Digester	H 13.83 m D 15 m V 2380 m^3^	Draft Tubes	Wastewater Sludge	3 D	Hexahedral 359900	Yes	L	Single Phase	k-ɛ Standard	Yes	Steady State	–	CCM+ (CD-Adapco) V. 6.06	Yes	[78]
Batch Reactor(s)	H 30 cm D 24 cm V 13 L	Self-Mixing	Cow Manure	2 D Axisymmetric	102560	Yes	L	Single Phase	Laminar	Yes	Time Dependent	–	COMSOL 5.5	No	[79]

∗ FS= Feedstock, GS = Grid Study, HT= Heat Transfer, CT= Computational Time, MV= Model Validation NC = Not Clear, NR = Not Reported.

a Models explained in Section 3.1.

Similarly, Craig et al. [77] reported that RSM (Reynolds Stress Model) is a complex model which solves seven additional equation as compared to the two equation k-ω (SST) model with quite similar results, hence former is computationally expensive (Table 4). In comparison of two equation models, the k-ɛ (RNG) has been found to give more accuracy, however still needs more computational power [38,80]. Wu [44] studied the modelling strategy for gas mixing in AD reactors and recommended that the k-ω (SST) model is suitable to predict the core flows in the digestion vessel. This model was presented by Menter [81] by combining the Wilcox k-ω and the k-ɛ models. This combined model has ability to solve the turbulence flows with more accuracy and validity [82]. Based on less complexity and need of computational power, the k-ɛ model was used in different studies [58,60,76,78,83,84] for the modelling of turbulence flows in AD reactors (Table 3). Similarly, some of the researchers reviewed in the current study [36,[71], [72], [73]] assumed the flow in the reactors as laminar and hence the Laminar Flow model was used. However, it is important to consider that in the modern AD reactors like UASB, EGSB etc. the flow has been calculated to be higher Reynolds number particularly in the baffled regions where recirculating flows are generated with negative vertices and wakes in the domain, resulting in localized turbulence flow trends (Table 4).

### 3.2 Modelling of multiphase behavior

**Fig. 9:**
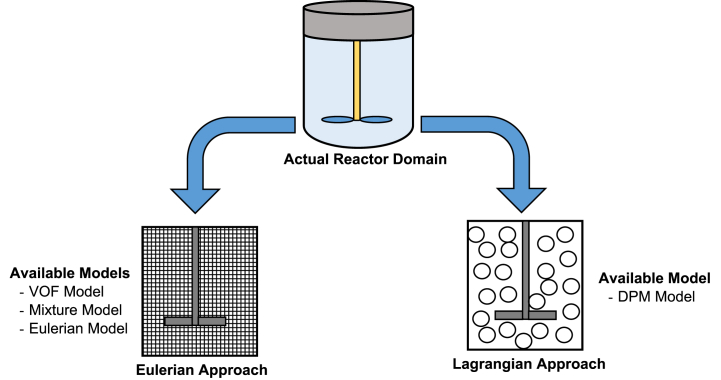
Eulerian and Lagrangian approaches in CFD modelling of AD process [16,89].

Typical multiphase phenomena are found in AD reactors as illustrated in Fig. 9. The presence of solid, liquid and gas phases make the AD process more complex which needs to be classified for better modelling and simulation. Considering the existing of multiple phases in closed domain, two computational approaches are generally used to model the multiphase phenomena in fluid flow systems, i.e., Eulerian-Eulerian (E-E) approach and Eulerian-Lagrangian (E-L) approach (Fig. 9). In the former approach, the secondary phase (fluid) is considered as continuum and solves the mass/momentum equations in the control volume same as the first/primary phase. Under the Eulerian frame of reference, the transport equation for the multiphase momentum transfer is given by Refs. [38,64,75]:

(8) $$\frac{\partial }{\partial t}\left(\alpha_{q}\rho_{q}{\vec{v}}_{q}\right)+\nabla .\left(\alpha_{q}\rho_{q}{\vec{v}}_{q}{\vec{v}}_{q}\right)=-\alpha_{q}\nabla P+\nabla .{\overset{═}{\tau }}_{q}+\alpha_{q}\rho_{q}\vec{g}+\sum_{p=1}^{n}\left({\vec{R}}_{pq}+{\dot{m}}_{pq}{\vec{v}}_{pq}-{\dot{m}}_{qp}{\vec{v}}_{qp}\right)$$

where P is pressure shared by all three phases, ${\overset{═}{\tau }}_{q}$ is the stress strain factor of the qth phase, ${\vec{R}}_{pq}$ represents the interaction forces between different phases whereas interphase velocity is indicated by ${\vec{v}}_{pq}$.

On the other hand, E-L approach considers and tracks the path of every individual particle also termed as control mass approach. Hence, this method is applied in case of high concentration of dispersed particles present in the secondary phase. It has also been found that with the increase in concentration of secondary phase particles, the computational requirement also increases [18,[85], [86], [87]]. The governing equation for the computation of momentum balance between the fluid phase and disperse phase (particles) is given by Refs. [64,82,88]:

(9) $$m_{p}\frac{d\vec{u_{p}}}{dt}=m_{p}\frac{18\mu_{f}}{\rho_{p\;d_{p}^{2}}}\frac{C_{d}R_{e}}{24}\left(\vec{u_{f}}-\vec{u_{p}}\right)+m_{p}\frac{g(\rho_{p}-\rho_{f)}}{\rho_{p}}+\vec{F_{s}}$$

Where, $m_{p}$, $u_{p}$ , $d_{p}$ and $\rho_{p}$ represent mass, velocity, diameter and density of the discrete phase (particles), respectively whereas $u_{f}$ is the fluid velocity, $\rho_{f}$ is the fluid density, $\mu_{f}$ is the fluid viscosity, $C_{d}$ is drag coefficient and $F_{s}$ denotes the external body forces.

Table 5 summarizes the application of various models under E-E and E-L approaches and their applications.

**Table 5:** Selection of multiphase models [16,90,91].

Multiphase Approach	Models	Application
Eulerian-Eulerian Approach	VOF Model	Interface between multiple immiscible fluids Examples: Free surface flows, Large bubble flow
	Mixture Model	Multiphase fluids with different velocities Simpler version of full Eulerian model Widely distributed dispersed phase in the whole volume Computationally less expensive Examples: Particle-laden flows, Sediment transport, slurry flow
	Eulerian Model	Dispersed phase concentrated in a part of the whole volume Interfacial drag law applications More accurate than Mixture model Example: Particle fluidization, Flow with suspended particles, Sediment transport and a wide range of solid (particle)-liquid mixing
Eulerian-Lagrangian Approach	Discrete Phase Model (DPM)	If secondary/dispersed phase volume fraction is ≤ 10 %. Volumetric fraction of secondary phase can be negligible. Tracking of individual trajectories at specific internals Examples: Spraying, particle injections to the flow field

The following approaches are used by the researchers to simulate the AD reactors:

- Single Phase L (with given TS concentration)
- Two Phase L/S (interaction of liquid substrate and sludge particles)
- Two Phase L/L (Co-digestion of different substrates)
- Two Phase L/G (interaction of liquid substrate and biogas bubbles)
- Three Phase L/S/G (interaction of liquid substrate, sludge and biogas bubbles)

A simplified approach used to simulate AD reactor is to assume the flow field in the reactor as single phase based on fluid properties as studied by various authors reported in Table 4. Wu [67] studied the AD reactor by assuming manure slurry as a single phase while neglecting the interaction between gas-liquid. Agborambag et al. [73] presented a CFD model for the simulation of the Chinese Dome Digester by considering liquid slurry as a single phase. Saini et al. [76] considered wastewater as homogenous and incompressible non-Newtonian phase in the reactor domain. Similarly [68,72,78], also employed single phase approach to study the mixing in AD reactors (Table 4). However, non-Newtonian behaviour as well as effect of total solids (TS) concentration in the liquid phase can be investigated using the Power Law Model [37,39,69,77]. In this case, the mixing tends and dead volume can be approximated while effect of different phases on hydrodynamics of the reactor is compromised. Hence, results produced in this way can only be used to find mixing speeds for mixers and agitators.

Two phase modelling using Eulerian-Eulerian approach has been found a step forward way to meet such challenges and predict the hydrodynamics of AD reactors in more realistic manner. Two approaches are used under this technique, i.e., liquid-solid and liquid-gas. The adoption of either approach depends greatly on the reactor design configuration and expected process output (Table 5). For instance, Zhang et al. [40] investigated the co digestion of cattle manure and corn stover (both were diluted in water to form a liquid slurry) using a two-phase mixture model. The corn stover was termed as primary phase while cow manure was considered to be the secondary phase in the mixture setting the co digestion ratio with the help of ‘Patch Function’ in the ANSYS (Fluent).

Two Phase L/G approach was also used by different researchers reviewed in the current study. D'Bastiani et al. [18] presented a CFD study to simulate UASB reactor validated with PIV experiments. They used Eulerian-Eulerian approach for the two phase, i.e. liquid/gas modelling. To describe the AD process, primary phase was considered to be water while dispersed phase (secondary phase) was labelled with the mixture of methane (65 %) and carbon dioxide (35 %). It was argued that this approach is more advantageous as compared to the Eulerian-Lagrangian approach in terms of computational complexities. Moreover, the main source of momentum transfer is the drag force between the fluid particles. Similarly, Li et al. [75] also used the L/G approach earth Eulerian approach for the AD reactor where food waste was considered as primary phase and gas was taken as the dispersed phase in the domain. It is important to note that the velocity of both phases might be different, however, turbulence parameters such kinetic energy and the dissipation rate remain constant under this approach [75]. Similarly Shreshta and Lohani [71], Wu [44] and Rasouli et al. [38] also reported CFD strategies with multiphase phenomena of liquid and gas as primary and dispersed phase, respectively. Latha et al. [70] investigated the mixing of AD reactor by injecting hydrogen gas with high pressure (Table 4). Being disperse phase, hydrogen gas was injected with Lagrangian method which can be done with the help of discrete phase model (DPM).

However, it is important to consider the combination of the three phases for more realistic simulations of AD reactors, particularly for the case of granular reactors like up flow reactors containing granular sludge particles [18]. It has been observed that the interaction of three phases in the domain occurs because of three major factors, i.e. drag, lift and mass making the solution more complex and computationally expensive [74]. Among the paper reviewed, a few researchers have tried to solve these phases within single computational domain to accurately predict the true flow fields in AD reactor. For example, Wang et al. [58] reported a CFD study by considering three phases, i.e. wastewater (L), sludge (S) and hydrogen gas (G) all as an individual continua in an EGSB reactor. Liquid phase, i.e., wastewater was taken as primary phase while solid and gas phases were considered as secondary phases in the reactor. Being a complex phase interaction, a 2-D domain was studied. Another research was presented by Pan et al. [49] in which two dimensional EGSB domain was studied with three phases, i.e. wastewater, sludge and biogas. Effect of baffle arrangement was studied for the optimization of baffle arrangement in three phase separation zone. Under this concept, the washing of solid particles is a critical factor which can be simulated with respect to different up flow velocities, sludge particle sizes, sludge density, bubble size of gas phase and wastewater initial composition with suspended solids.

### 3.3 Modelling of heat transfer

Generally, two kind of heat flows are experienced by the AD reactors, i.e., 1- external heat transfer to reactor to maintain the required temperature of substrate and 2- fluctuation in hydrodynamic behaviour because of heat of reaction. For an optimum and stable AD process, it is very essential to keep and maintain the temperature of AD reactor at a required level and hence is considered as priority while designing an AD reactor. Due to fluctuation in ambient temperature at lab scale as well as at industrial scale, external heating systems are provided to meet such requirements. At lab scale, the required temperature is maintained with the help of water bath and heating coils while large boilers and heat exchangers are used for the industrial scale AD systems. Most of such heating systems are operated either by electricity or auxiliary fuels which increases the operational cost for the AD plant [52]. This is even critical for the cold climate regions where temperature drops to very low level resulting in extensive energy input. Therefore, it is necessary to find sustainable ways to provide heat supply to the AD reactor [52,92].

Heating of AD reactor can be configured in two ways, i.e., direct heating and indirect heating. In direct heating, a jet of hot steam or water is injected in the AD reactor which heats up the organic matter. However, this method faces several technical problems such as overheating, high energy requirements and dilution of substrate etc. On the other hand, hot jet of steam or other fluid is used to heat up the reactor indirectly, i.e., without being in contact with the organic matter. There are different designs and configuration of such heat exchangers which can be used for AD reactor such as floor heating system, solar heater (built-in heating chamber), in-vessel heating tube and exhaust gas recirculation etc. Another approach is heating through electromagnetic radiation, i.e., microwave with a wavelength of 1 mm-1m and a frequency of ranging from megahertz to gigahertz is passed through the reactor domain. This leads to volumetric heating of the organic solution without uneven heating of reactor boundaries, leading to efficient digestion process [93,94]. However, selection of either type of heat exchanger depends upon the shape and size of AD reactor [95]. A modelling of heat flux in a heat exchanger used in AD reactor has been presented in Ref. [95] which is however based on qualitative heat loss. Similarly, other methods of heating the AD reactor include use of geothermal energy as a geothermal heat pump, underground water heat pump, solar thermal collector and circulating thermal fluids etc. [[96], [97], [98], [99], [100], [101], [102], [103], [104], [105]], however it is always necessary to model the efficiency of heating system for sustainable AD process. CFD modelling approach with coupled heat balance is used to simulate the heat flow from heat exchanger to the reactor for stabilization of AD process. In this context, modelling of heat flow through the reactor walls and insulation of reactor domain is help to find the correct material composition used to develop the AD reactor. The heat transfer is based on both conductive as well as convective heat flows in AD process and hence can be studied by incorporating the heat transfer through walls of the reactor domain in CFD codes, i.e., ANSYS (Fluent). The shear forces are also coupled with the heat flows of the fluid. For this purpose, the temperate of reactor walls is defined to as per the expected heat flux in the real systems. Similarly, the adjacent fluid zone is also coupled for the convective heat flux. The temperature distribution obtained from the CFD results is used as a decision tool for the reorientation of AD reactor configuration. From the extensive review, it has been found that most of the researcher have modelled hydrodynamic and mixing in reactor while very few studies have been found in relation with heat transfer to the reactor. For example, Perrigault et al. [106] presented a one dimensional mathematical model for the analysis of heat transfer from solar collector to the anaerobic digester. Based on simplified approach, a further two dimensional model was proposed to include effect of air inflow and wall heat flux. Similarly, Chen et al. [107] and Chen et al. [108] studied different geometrical shapes of heat exchangers for the thermal treatment and heating of AD reactors. It was recommended to optimize the AD reactor with heat exchanger tubes for best adaptability at large scale. Wu and Bibeau [109] presented a three dimensional heat transfer model for the modelling of heat transfer in AD reactors for cold climate regions. From different geometrical configuration of the reactor insulation walls, flat shaped top wall was found to sustain heat more effectively. Ibrahimi et al. [92] investigated CFD modelling of heat transfer to organic substrate through liquid recirculation and found that high liquid recirculation rates contribute to better heat transfer to the substrate inside the reactor, however, it needs to be optimized with hydraulic efficiency of the reactor.

Like other biochemical processes, AD involves chemical reactions with different heat of reactions. For example, the first two phases of AD process, i.e., hydrolysis and acidogenesis are exothermal processes in which heat is liberated inside the reactor while the later ones (acetogenesis and methanogenesis) are endothermic phases. This fluctuation in temperature results in natural convective circulation within the AD reactor which also effect the internal fluid hydrodynamics. Hence, it is also necessary to consider the heat flow within the reactor for realistic prediction of AD process [79] which is modelled by coupling reaction kinetics in CFD code. Based on the current review, it has been found that very limited information is available on the heat transfer of AD reactor coupled with other process characteristics, hence there is a gap in the research to study and present a full scale CFD model for the computation of heat transfer to and from the AD reactor with modern configuration such as up flow rectors in line with the other AD performance parameters.

### 3.4 Coupling of species kinetics (bio-kinetics)

The organic matter (i.e., feedstock) undergoes biochemical destruction in the AD reactor. Hence, bio-kinetics is one of the most important processes of anaerobic digestion. As described in section 3, ADM1 [59] has been the most widely used model for the prediction of AD process. This model assumes the uniform mixing of the substrate in the reactor volume. The change in concentration of each species is therefore, given by Refs. [59,110,111]:

(10) $$\frac{dS_{i}}{dt}=\frac{Q_{in}S_{i,in}}{V}-\frac{Q_{out}S_{i}}{V}+\sum_{j}\rho_{j}.v_{i,j}$$

where, $Q_{in}$ and $Q_{out}$ is the flow rate of substrate at inlet and outlet, $S_{i}$ and $S_{i,in}$ represents the concentration of the component in the reactor and at the inlet, respectively, V is the reactor volume of the reactor. The term $\sum_{j}\rho_{j}.v_{i,j}$ is the sum of kinetic rates as described by Batstone et al. [59].

Although it is a comprehensive model, it is based on the assumption of completely mixed/stirred AD reactor. Because of various constants and coefficients used in this model, it is sometime difficult to compare and validate the model with experimental data [112]. Coupling of bio-kinetics models with hydrodynamic approaches in CFD can provide a more realistic results as compared to the conventional models. However, it is still challenging to couple such models for the optimization and improvement of AD reactors [18]. Rezavand et al. [112] used gpuSPHASE framework approach [113] to couple CFD and reaction kinetics model using full Lagrangian method which tracks the individual particles so that biochemical balances can be solved for each particle in the domain. Similarly, Kumar et al. [110] has recently adopted differential algebraic equation to couple hydrodynamics with AD biokinetics. However, because of complex bio-reactions taking place in AD process, coupling of reaction kinetics with CFD models remains a critical challenge [18]. Linking CFD with compartment modelling approach (also termed as multizone modelling) is another way to simplify the model complexities caused by coupling of mass and momentum transfer with biokinetics. In this technique, the reactor domain is divided into multiple interlinked zones where the hydrodynamics, flow field and biokinetics are solved and averaged for each individual volume-based compartment [114]. This approach enables studying the impact of fluid dynamics on biodegradation. However, underestimation and simplification of AD attributes lead to lower accuracy when moving from lab scale setup to industrial scale applications. Hence, full integration of ADM1 model parameters with the CFD-based compartmental models needs to be addressed in future studies [15,[115], [116], [117]].

### 3.5 Application of machine learning (ML) approach

Computational time required to produce reliable CFD results remains one of the major concerns while dealing with multi-physics problems with complex geometries. This becomes more crucial while dealing with time dependent problems where smaller time steps are inevitable to simulate the flow dynamics. For example, every instance has a specific attribute during anaerobic digestion (transient) process. This results in a high number of data sets requiring more time for post processing of CFD results. With the introduction of modern hardware, computational resources and robust algorithms, it has become relatively easier to process big data with the help of machine learning (ML) approaches. Different ML approaches such as supervised leaning, unsupervised learning and semi supervised/hybrid learning are employed over a wide range of applications. Under this concept, the ML algorithms are trained with the input data sets to predict a test output. As a result, the parametric investigations become easier with less computational costs. Such approaches are useful for the establishment of fundamental relationships between different process functions that could be used in process monitoring, control and optimization. Moreover, most of the CFD simulations are based on user defined assumptions to mimic the real process such as kinetic constants, particle/fluid properties, reaction rates etc. Due to human based assumptions, many important factors are likely to be ignored impacting the overall results. ML offers a more reliable assumptions which can be applied to the CFD models with greater accuracy. In this way a hybrid modelling approach can provide more realistic results with less time as well as computation cost [118]. However, there are certain constraints for coupling of CFD and ML in multiphase studies. For instance, availability of sufficient data to train the neural networks is one of the major challenges. Similarly, the flow fields may vary in time dependent cases which produce distinct data sets that needs to be pre-processed before employing in ML codes. Availability of open access data sets generated from CFD as well as experimental studies of various AD reactor formations would be helpful to find the fundamental correlations through ML approach [[118], [119], [120], [121]].

### 3.6 Validation and verification of CFD modelling results

From the above mentioned guidelines, a comprehensive CFD model can be developed for the simulation of desired AD reactor. However, it is always necessary to verify and validate the model with respect to actual performance. CFD results can be varied from the mass and energy balances and correlations obtained from ML approaches which could provide an estimation of error on the results. Reliability of CFD model to high level can be achieved by comparing the results with experimental data. When considering the AD process and multiphase problems, different experimental approaches are used such as Tracer Experiment Method, Particle Image Velocimetry (PIV), Pressure Analysis (through Manometers), Particle Tracking Experiments, shadowgraphy etc. which can produce data and images to be compared with CFD results, as reported by D'Bastiani et al. [18]. Radioactive tracer is another technique used to measure the residence time of the substrate in AD reactors with more accuracy as compared to the conventional tracers [122,123]. Scale down technique is also helpful tool for the confirmation of simulation results with less experimental cost [124,125]. The validation of a certain AD process depends upon the available resources and level of accuracy desired.

Although modelling and simulation of AD reactor depends upon the available computational resources and coding platforms, the simulation strategy using CFD includes common steps as: 1) Geometry formulation: the geometry and design of AD reactor (2D/3D) is formulated with the help of CAD tool, 2) Mesh generation: the reactor geometry is transformed into smaller computational cells or grids, 3) Solver and model application: the computational mesh is loaded into a solver platform which is used to apply and couple different sub-models (Section 3), 4) Provision of boundary and initial conditions: depending upon the type, composition, concentration and physical properties of the AD constituents, flow rates, inlet and outlet conditions, boundary layers and reactor walls etc., the boundary and initial conditions are provided, 5) Running the model iterations: the calculations are run for a specific time period and time steps, 6) Results processing and validation: the results are extracted and processed in the desirable format to compare with the experimental analysis (Table 4) [67].

## 4 Summary and future recommendations

Controlled digestion of organic constituents is essential to obtain maximum performance and high efficiency which can be achieved with an efficient AD reactor design. Computational fluid dynamics is a useful tool for the designing and testing of AD reactor under different operating scenarios. This review presents comprehensive guidelines for the design optimization of AD reactors by highlighting the linkage between physical AD processes and associated CFD modelling attributes. CFD modelling and simulation of AD reactor have been elaborated in line with all components in typical AD process. Due to presence of three phases in single domain, development of a comprehensive CFD model is challenging. Furthermore, it should be noted that the current study was focused mainly on the liquid state AD processes. Further investigations are recommended to study and compare the modelling of solid state process and its coupling with the proposed CFD models. The major aspects of the current review are summarized as below:

1. Reactor design and configuration is considered as a prime importance for AD process. Reactor hydrodynamic and phase interaction is significantly affected by the geometrical shape of the reactor. Modern up flow AD reactors, i.e., UASB and EGSB etc. give a greater process efficiency, however it is necessary to find the optimum design of three phase separators and operating parameters, i.e., up-flow velocity, particle size, sludge bed etc. which can be studied through multiphase CFD simulation. Sufficient retention of sludge particle is a major concern for up flow reactors which should be further investigated through multiphase CFD studies to find improved design configurations which can ensure the retention of sludge particles.
2. Mixing of the organic substrates is mostly carried out with the help of mechanical agitators which is an energy intensive process. Mechanical mixing and agitation with respect to position, speed, frequency as well as rotational direction must be optimized using CFD modelling to minimize mechanical energy requirements. The mixing of the substrates through gas transfer as well as hydraulic recirculation is suitable for low TS feedstock such as wastewater especially in fluidized reactors. However, high turbulence in the reactor results in washing out of biomass/sludge particles from domain, hence optimization of both design and process configuration is important in such scenario. The k-ɛ model and k-ω model are commonly applied for modelling of AD process. Whereas k-ɛ (Realizable) model is suitable for high-rate AD reactors while the k-ω (SST) is more suitable for the AD reactors with mechanical mixers. However, with the introduction of modern parallel computing facilities, the RSM model should also be further explored, particularly, in case of hydraulic and pneumatic recirculation.
3. Most of the CFD studies have assumed the reactor fluid as a single phase, however, three phases (solid, liquid and gas) are identified in a typical AD process which has a significant influence on organic matter degradation as well as hydrodynamics in the reactor. Some studies have reported multiphase modelling using two phases either liquid-solid or liquid-gas while the effect of third phase is still ignored. Incorporation of all three phases is challenging which needs to be examined. Combined momentum caused by gas bubbles, sludge particles and buoyancy forces of liquid phase need to be investigated. Similarly, implementation of Lagrangian approach is also desirable while dealing with the feedstock substrates with more dispersed particles for the tracking of solids as well as gas bubbles in the reactor and hence discrete phase model (DPM) should be coupled with the other fluid flow models for more realistic results. While more reliable predictions can be obtained with such advanced models, at the same time, more computational resources are required. In this context, sensitivity analysis can help to identify the most as well as the least influential process parameters. As a result, the least sensitive components can be simplified in the model whereas the highly sensitive indicators affecting the simulations results at large need more attention. Moreover, desirable level of output and accuracy is also worthwhile to decide the corresponding modelling strategy, i.e., single phase or multiphase (two phase/three phase) approach.
4. Breakage and agglomeration of sludge particle are also important phenomena which affect microbial count in the AD reactor. This needs to be investigated, e.g., through population balance modelling (PBM) to ensure particle concentration required for AD process.
5. Heating of AD reactor always remains a critical aspect as it effects the bacterial growth and degradation activities. Most of the AD reactors are heated with electrical heater or water bath system. In order to provide sustainable approach for the heating to maintain a constant temperature of AD reactor, it is essential to study the conductive as well as convective heat transfer through walls and the flow regime using CFD models. Heat losses from the reactor walls should also be studied for the selection of appropriate thickness of the reactor walls. Moreover, other heating applications such as solar thermal collectors, thermal oils recirculation, microwave heating and geothermal heat pumps should also be studied through CFD simulations for sustainability of the overall AD system. Moreover, heat of reaction should also be considered as it results in change in reactor temperature during the four AD phases effecting the reactor hydrodynamics.
6. Coupling of biochemical reactions and species kinetics remains one of the biggest challenges in CFD modelling of AD processes. The ADM1 model and its extensions for AD process needs to be coupled with the CFD model to describe the biochemical destruction of the organic matter and resulting flow trends due to gas phase interaction. This may increase the model complexity and computational time which can be avoided by using a simpler modelling strategy such as 2D or axisymmetric reactor domain without compromising the accuracy of the results.
7. Successful coupling of CFD with ML models to formulate a hybrid prediction model for AD process is still in early stage which need to be studied for AD process. This approach would be helpful to introduce fundamental relationships and correlations for process parametric optimization as well as identification of CFD settings. Development and analysis of robust ML algorithms for AD process needs to be carried out in context of CFD outcomes of AD process.
8. Verification and validation of the simulation codes is necessary to achieve degree of accuracy and reliability of results, hence, developed CFD models should be compared with the empirical correlations as well as experimental findings for successful adoption. In this regard, the scale down technique is found to be helpful to minimize the experimental costs. However, the validation of a certain AD process depends upon the available resources and level of accuracy desired.
9. Besides treatment of large volume of organic wastes, AD process results in significant energy potential in terms of biogas which can be used in combined heat and power (CHP) systems. CFD simulation of AD reactors can be integrated as a short-cut model for the simulation and performance assessment of overall biogas production unit, enabling it as a sustainable energy technology. Integration of plant simulators and CFD needs to be further explored for optimization of industrial scale biogas plants.

## CRediT authorship contribution statement

**Muhammad Usman Farid:** Writing – original draft, Methodology, Investigation, Formal analysis, Conceptualization, Writing – review & editing. **Indiana A. Olbert:** Supervision, Methodology, Project administration. **Andreas Bück:** Writing – review & editing, Supervision, Methodology, Funding acquisition, Investigation, Resources. **Abdul Ghafoor:** Writing – review & editing, Investigation, Conceptualization, Formal analysis. **Guangxue Wu:** Funding acquisition, Methodology, Project administration.

## Ethical statement

This study did not involve any data collection from human or animal subjects.

## Data and code availability statement

Data included in the article/supplementary material is referenced in the article.

## Declaration of generative AI use

The authors declare that no artificial intelligence tool was used in the writing or editing process of this paper.

## Declaration of competing interest

The authors declare that they have no known competing financial interests or personal relationships that could have appeared to influence the work reported in this paper.
